# New Anti-Periodics

**Published:** 1853-11

**Authors:** 


*New Anti-Periodics.*—Dr. Holton recommends *Prairie Dock—Parthenium Integrifolium*—as a remedy for periodic fever. He considers a decoction of two ounces of the dried tops of the plant, the equivalent of twenty grains of quinine. This is placing a higher value upon its remedial powers than has been accorded to any other substitute for cinchona, or quinine, that has been discovered. It proved successful in thirty successive cases, some of which were severe; and without any unpleasant effect upon the nervous system. If these observations are confirmed and established by further experience, the remedy will be of immense value. The root of the *Yellow Jessamine—Gelseminum Sempervirens*—has also been recommended as a valuable substitute for quinine. It is said to be of this root that the "*electric febrifuge*" is made, which is now used pretty extensively in the South, as a nostrum. The efficacy of this remedy is certified to by the usual number of *respectable* names, including, of course, dignitaries of the church; and we have seen it—the Jessamine—highly extolled in several medical journals.—In all the accounts we have seen, however, it has been used in conjunction with such large quantities of quinine, as to preclude the possibility of forming a correct judgment of its anti-periodic efficacy ; and the same is true of the electric febrifuge. Whether the two remedies be identical or not, they are both powerfully narcotic, and it is probable their remedial virtues are due to this quality. But we have no reason to suppose them superior to the stramonium, and other narcotics which have been in use as febrifuges from time immemorial. A species of *Verbena*, we believe the *V. verticifolia*, has been successfully used as an anti-periodic in the neighborhood of this city. Several families which have been annoyed by intermittent fever, declare that they have found this herb commonly called vervain, or vervine—so efficacions as to supersede the use of quinine. They use the green herb itself, in decoction.—*Memphis Medical Recorder.*

